# Supplementary material for: Dormant origin firing promotes head-on transcription-replication conflicts at transcription termination sites in response to BRCA2 deficiency
Source: Nat Commun. 2024 Jun 3;15:4716. doi: 10.1038/s41467-024-48286-1 (PMC11148086; doi:10.1038/s41467-024-48286-1)
Supplement: Supplementary file 1 — Supplementary Information [file 41467_2024_48286_MOESM1_ESM.pdf]

Supplementary Information

Dormant origin firing promotes head-on transcription-replication conflicts at transcription termination sites in response to BRCA2 deficiency

Liana Goehring[1], Sarah Keegan[1,2], Sudipta Lahiri[1,3], Wenxin Xia[1], Michael Kong[1], Judit Jimenez-Sainz[3], Dipika Gupta[1], Ronny Drapkin[4,5], Ryan B. Jensen[3], Duncan J. Smith[6], Eli Rothenberg[1], David Fenyö[1,2], and Tony T. Huang[1]*

[1]Department of Biochemistry & Molecular Pharmacology, New York University School of Medicine, New York, NY, USA

[2]Institute for Systems Genetics, New York University School of Medicine, New York University School of Medicine, New York, NY, USA

[3]Department of Therapeutic Radiology, Yale University, New Haven, CT, USA

[4]Penn Ovarian Cancer Research Center, University of Pennsylvania, Perelman School of Medicine, Philadelphia, PA, USA

[5]Basser Center for BRCA, Abramson Cancer Center, University of Pennsylvania, Perelman School of Medicine, Philadelphia, PA, USA

[6]Center for Genomics and Systems Biology, Department of Biology, New York University, New York, NY, USA

*Correspondence to Tony.huang@nyumc.org

This File includes:

Supplementary Figures 1-7

a

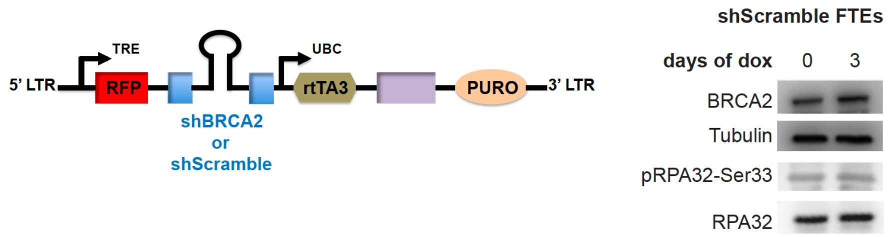

b

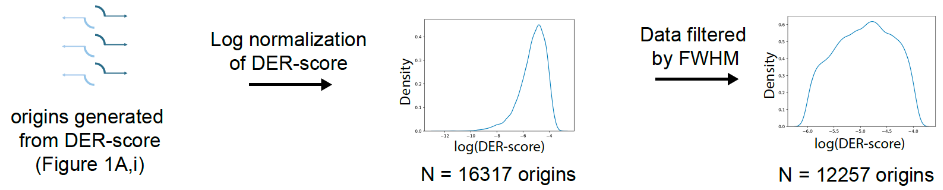

c

Cell line	Ok-seq sample	# of origins
RPE	Untreated	12,257
RPE	0.2mM HU	11,309
FTE194	0d-rep1	13,790
FTE194	0d-rep2	14,136
FTE194	3d-rep1	13,093
FTE194	3d-rep2	13,026
FTE194	6d-rep1	12,012
FTE194	6d-rep2	13,138

d

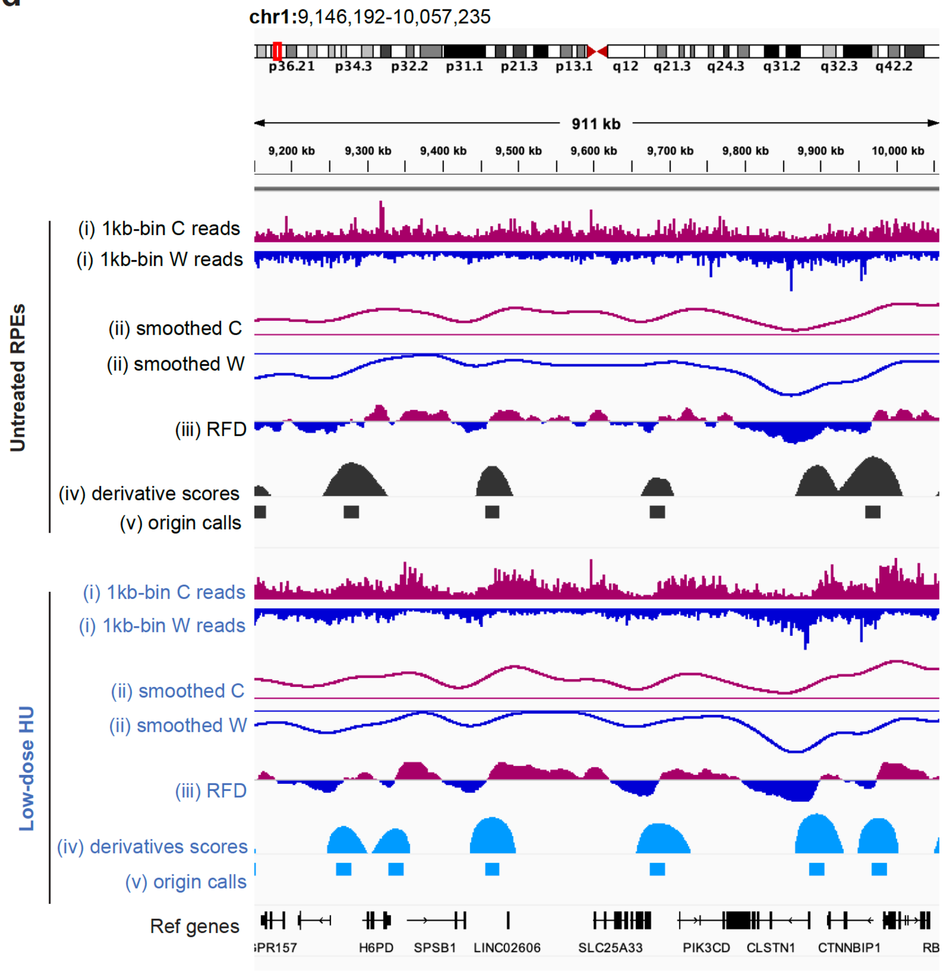

Supplementary Figure 1

Supplementary Fig. 1. Ok-seq origin call analysis. a (Left) Schematic of lentiviral TRIPZ shRNA expression construct. FTE (fallopian tube secretory epithelial) cells were stably transduced with a DOX-inducible shRNA targeted against shBRCA2 or shScramble in this study. (Right) Whole cell lysates of immortalized shScramble-FTEs after 0 or 3 days of doxycycline treatment were analyzed by western blot. **b** Origin filtering following DER_SCORE origin analysis. Origins generated from DER_SCORE origin analysis are log-normalized showing negatively skewed data. Full-width half-max normalization (FWHM) is used to isolate efficient origins. **c** Number of replication origins identified by Ok-seq origin analysis for each replicate of RPE-1 cells or FTEs. Ok-seq analysis of untreated or 0.2mM HU-treated, 4h RPE-1 cells analyzed from previously published Ok-seq data (Chen et al. 2019). shBRCA2-FTEs were analyzed by Ok-seq for replication origin analysis after 0, 3, or 6 days of knockdown. **d** Origin call analysis pipeline visualization. Representative locus showing (i) 1-kb binned Watson and Crick Ok-seq read abundance profiles, (ii) Hann smoothed Watson and Crick reads, (iii) replication fork directionality calculated as (C-W)/(C+W), (iv) DER-SCORE calculated from increasing DER(Crick) and decreasing DER(Watson), (v) origin calls retained after normalization and FWHM filter, each for untreated RPE Ok-seq sample (black) and HU-treated RPE Ok-seq sample (blue). hg19 refseq genes shown.

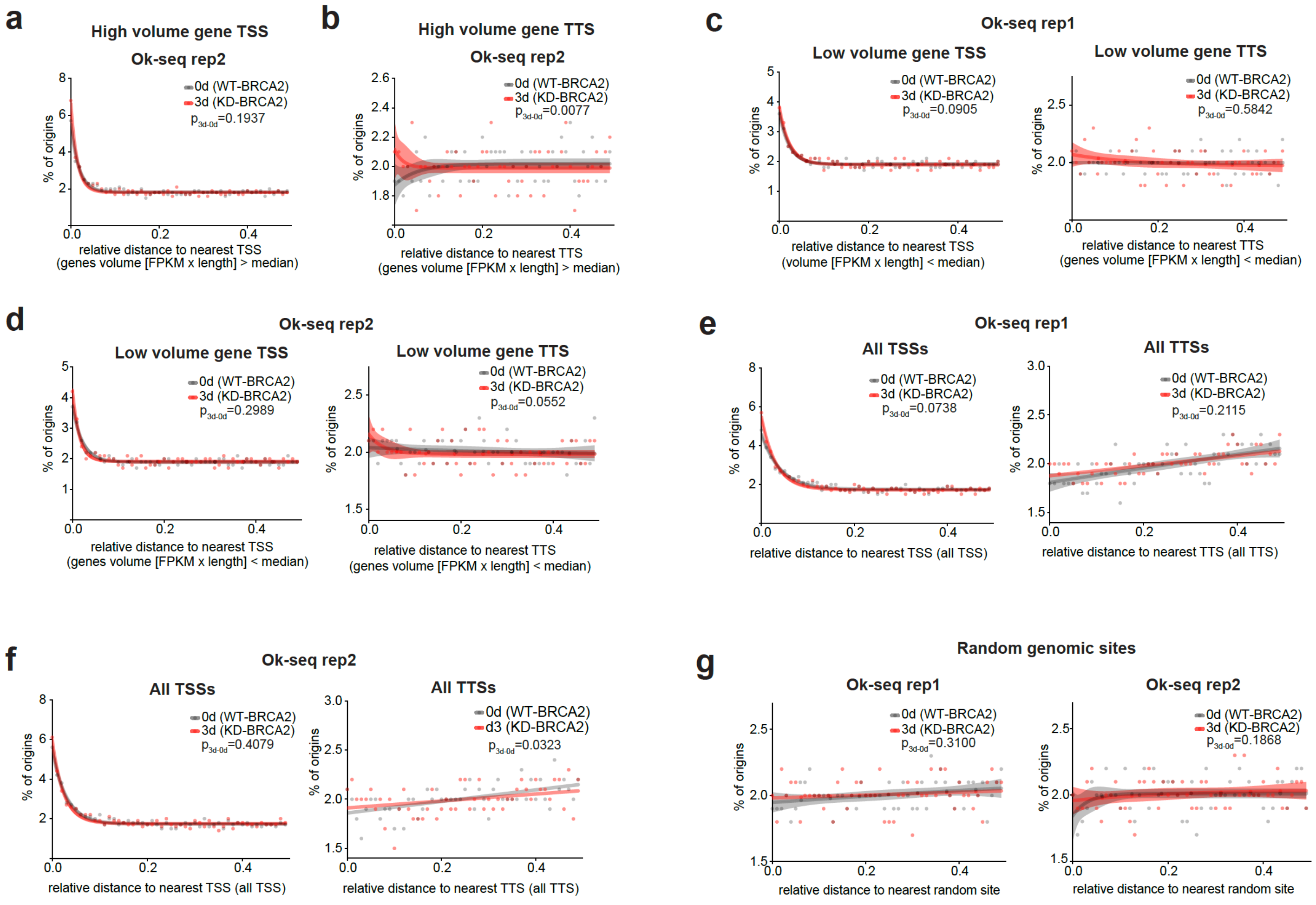

Supplementary Figure 2

Supplementary Fig. 2. New origins at TTSs of high-volume genes in BRCA2-KD FTEs by Ok-seq. a-b Second replicate of relative distance origin analysis of shBRCA2-FTEs relative to TSSs (**a**) and TTSs (**b**) of high-volume genes. **c-f** Relative distance origin analysis of two replicates of shBRCA2-FTEs relative to TSSs (left) and TTSs (right) of low-volume genes **(c,d)**, or all genes **(e,f)**. **g** Relative distance origin analysis of two shBRCA2-FTE Ok-seq replicates relative to random genomic sites.

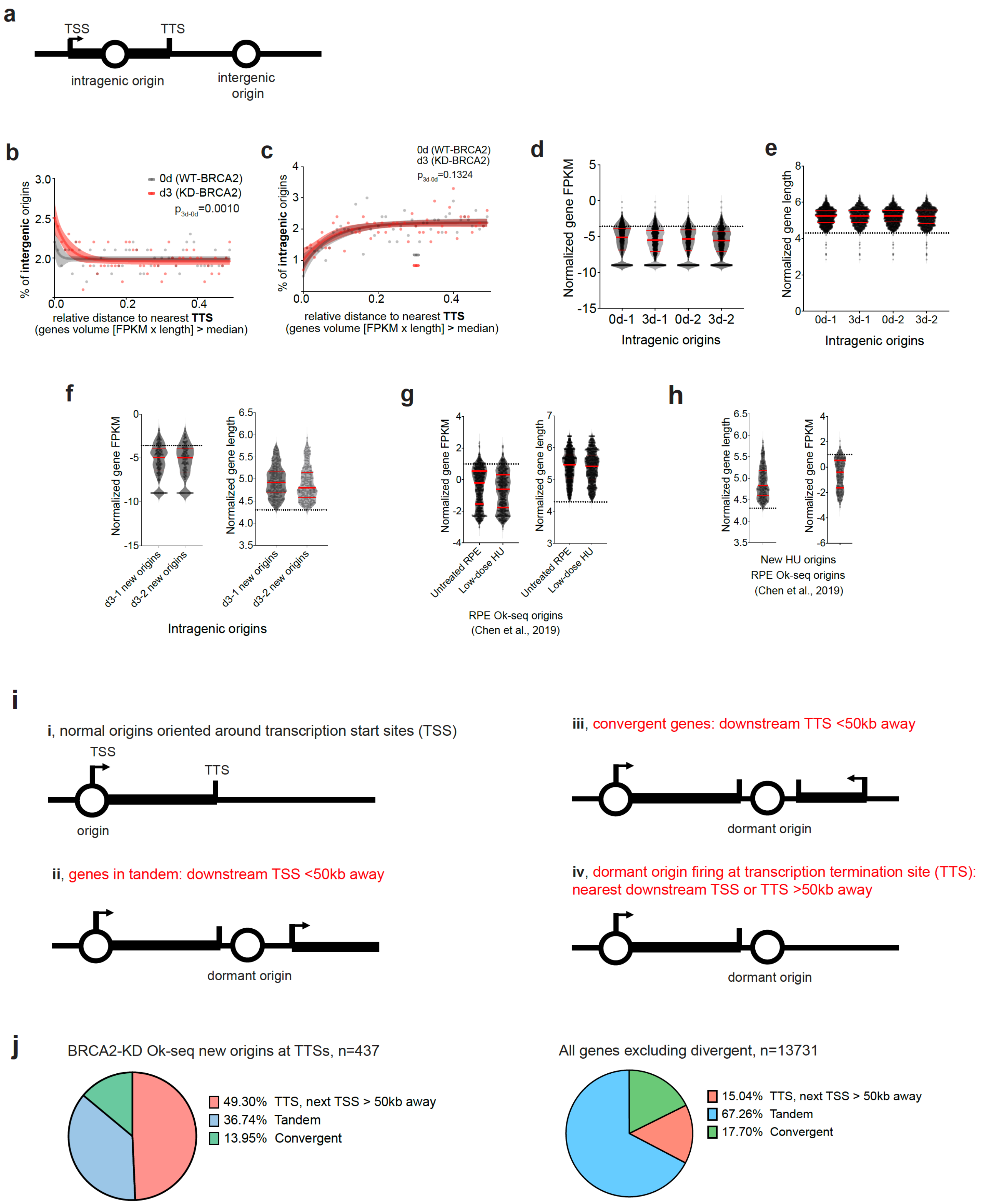

Supplementary Figure 3

Supplementary Fig. 3. Intra- and intergenic origin firing in BRCA2-KD FTEs. a Schematic of intragenic and intergenic origin firing. **b-c** Relative distance analysis of FTEs +/- shBRCA2 of either intergenic origins (**b**) or intragenic origins (**c**) relative to high-volume gene TTSs. **d-e** Log_{10} normalized gene FPKM **(d)** and gene length **(e)** of two biological Ok-seq replicates of untreated and shBRCA2-KD FTE intragenic origins (0d-rep1: N=1337, 3d-rep1: N=1081, 0d-rep2: N=1196, 3d-rep2: N=1029). Dotted line represents average FPKM and gene length (n=18250 genes from RNA-seq analysis of FTEs). **f** New intragenic origins in BRCA2-KD by two Ok-seq replicates characterized by FPKM (left) and gene length (right). 3d-rep1: N=258, 3d-rep2: N=203. Dotted line represents average FPKM and gene length (n=18250 genes from RNA-seq analysis of FTEs). **g** Log_{10} normalized gene FPKM (left) and gene length (right) of two biological Ok-seq replicates of untreated and HU-treated RPE-1 intragenic origins from Chen et al. (2019). (UNT: N=1630, HU: N=1238). Dotted line represents average FPKM and gene length (n=18250 genes from RNA-seq analysis of RPE-1 cells). **h** New intragenic origins in HU-treated RPE-1 cells by Ok-seq characterized by FPKM (left) and gene length (right). N=323. Dotted line represents average FPKM and gene length (n=18250 genes from RNA-seq analysis of RPE-1 cells). **i** Schematic of intergenic origin firing: i, canonical origins fire at TSSs of long and highly-transcribed genes in unperturbed cells; ii, dormant origin firing at TTSs where the nearest downstream TSS is less than 50kb away (genes in tandem); iii, dormant origin firing at TTSs where the nearest downstream TTS is less than 50kb away (convergent genes); iv, dormant origin firing at TTSs where the nearest downstream TSS or TTS is more than 50kb away. **j** Left: Characterization of candidate genes with new origins at TTSs in shBRCA2-KD FTEs. Right: Distribution of all genes excluding divergent as divergent does not characterize a gene's TTS (N=13731).

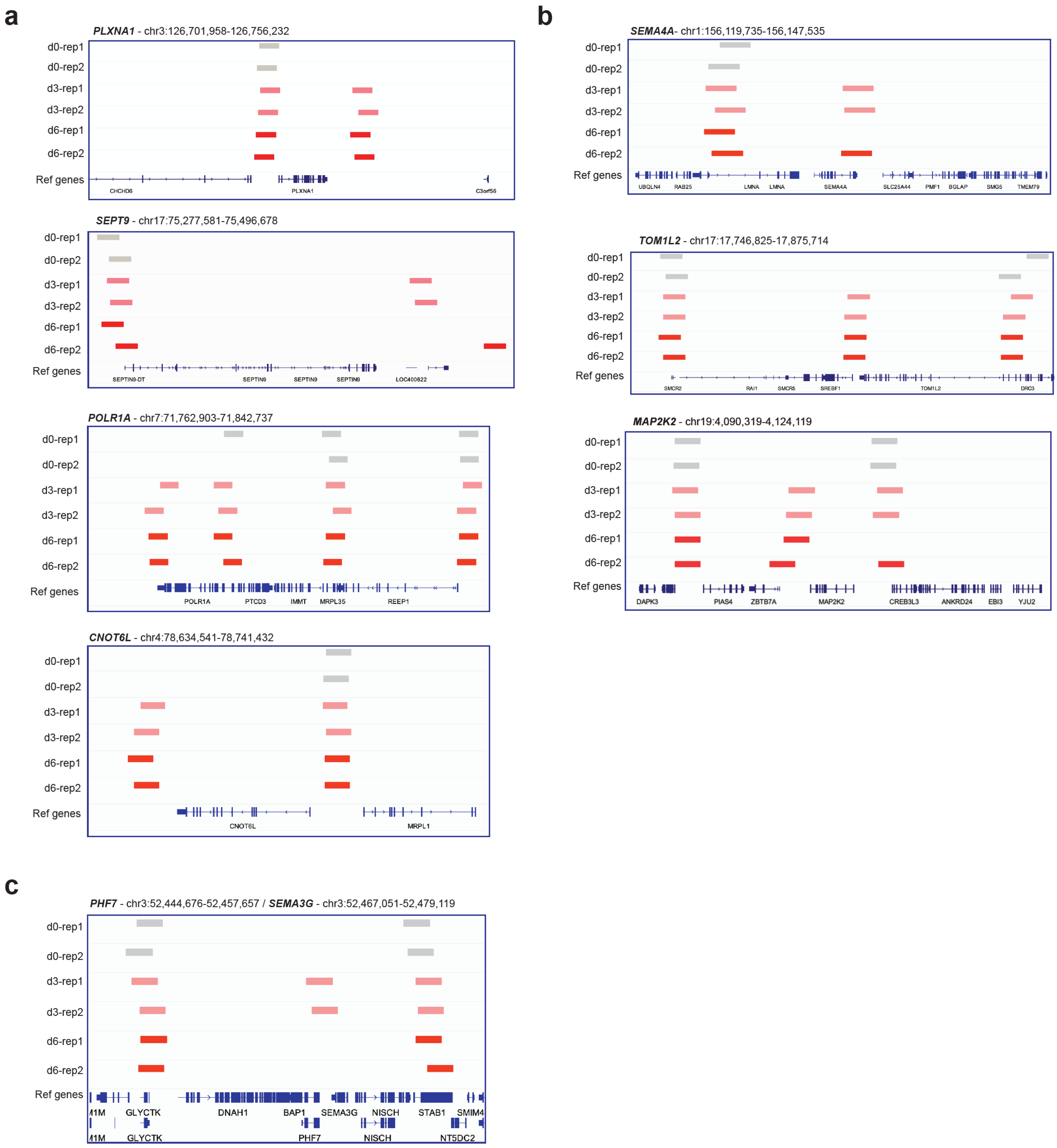

Supplementary Figure 4

Supplementary Fig. 4. Representative candidate genes. a Four representative candidate genes where the nearest downstream TSS or TTS is more than 50kb away. **b** Three representative candidate genes in tandem. **c** One representative candidate convergent gene.

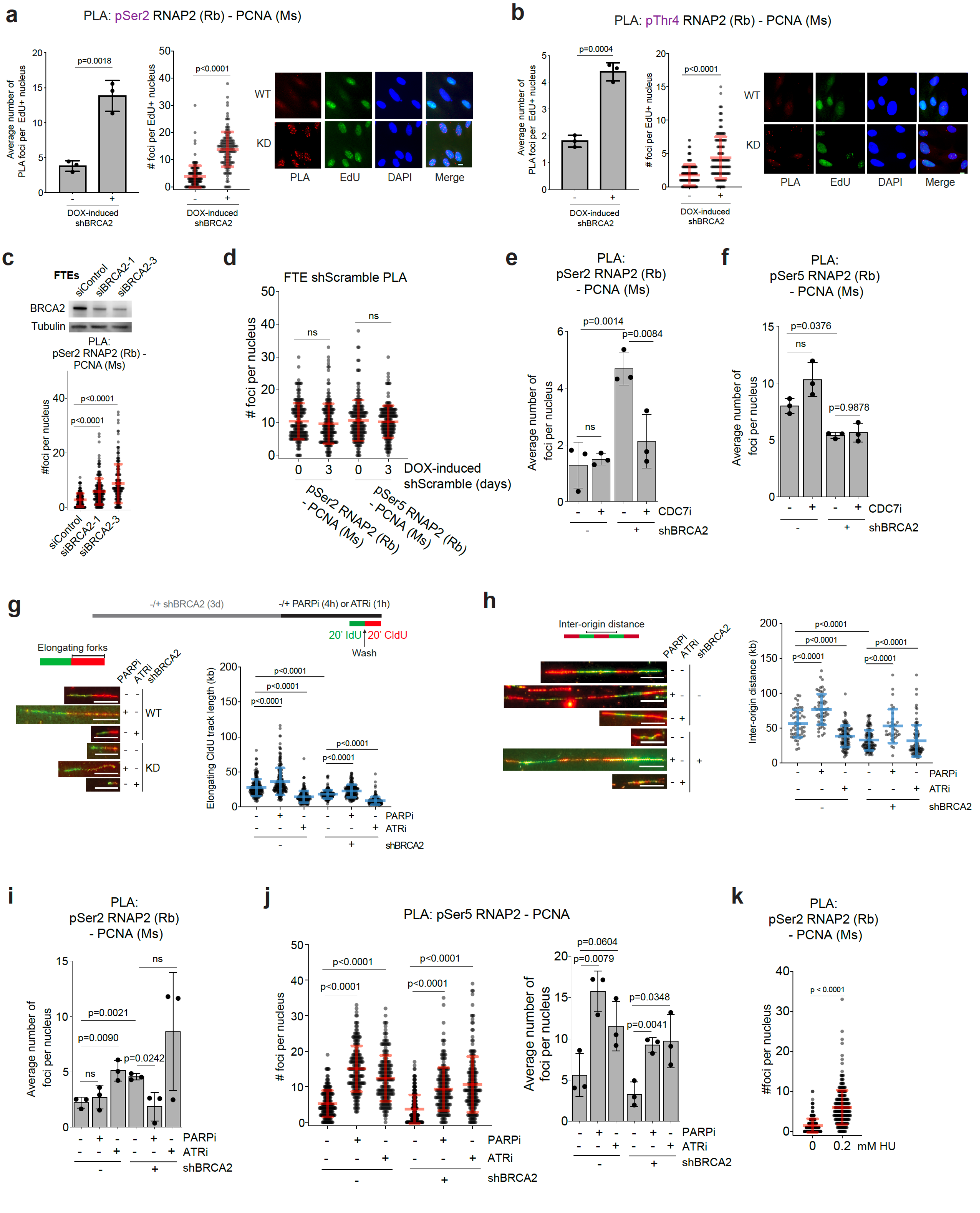

Supplementary Figure 5

Supplementary Fig. 5. BRCA2-KD causes HO-TRCs by PLA. **a** PLA of pSer2-RNAP2 and PCNA of pulse-labeled EdU+ FTEs +/- shBRCA2. Data presented shows 150 nuclei from 3 biological replicates. *p* values calculated using unpaired two-tailed t-tests. Left: average number of foci per nucleus per biological replicate. Right: number of foci per nucleus. Error bars = mean, std. Scale bar = 10μm in representative images. **b** PLA of pThr4-RNAP2 and PCNA of pulse-labeled EdU+ FTEs +/- shBRCA2. Data presented shows 150 nuclei from 3 biological replicates. *p* values calculated using unpaired two-tailed t-tests. Left: average number of foci per nucleus per biological replicates. Right: number of foci per nucleus. Error bars = mean, std. Scale bar = 10μm in representative images. **c** PLA of pSer2-RNAP2 and PCNA in siControl FTEs and FTEs treated with two siRNAs against BRCA2. *p* values calculated using unpaired two-tailed t-tests of 200 nuclei per condition from 3 biological replicates. Error bars = mean, std. **d** PLA of pSer2-RNAP2 and pSer5-RNAP2 each against PCNA in untreated and DOX-treated shScramble FTEs. *p* values calculated using unpaired two-tailed t-tests of 200 nuclei per condition from 3 biological replicates. Error bars = mean, std. **e** PLA of pSer2-RNAP2 and PCNA in WT- and KD-BRCA2 +/-CDC7i. Data presented shows 200 nuclei from 3 biological replicates. Average number of foci per nucleus per biological replicate, *p* values calculated using unpaired two-tailed t-tests. Error bars = mean, std. **f** PLA of pSer5-RNAP2 and PCNA in WT- and KD-BRCA2 +/-CDC7i. Data presented shows 200 nuclei from 3 biological replicates. Average number of foci per nucleus per biological replicate, *p* values calculated using unpaired two-tailed t-tests. Error bars = mean, std. **g** DNA fiber analysis measurement of fork elongation in FTEs. Representative images shown of DNA fibers from WT- and KD-BRCA2 +/- 10μM PARPi, 4h or 10μM ATRi, 1h. Scale bars = 10μm. Scatter plot shows quantification of elongating CldU track length. *p* values calculated using unpaired two-tailed t-tests of 200 fibers per condition from three biological replicates. Error bars = mean, std. **h** DNA fiber analysis of inter-origin distance in FTEs. Representative images shown of DNA fibers from WT- and KD-shBRCA2 +/- PARPi or ATRi. Scale bars = 10μm. Scatter plot shows quantification of inter-origin distance. *p* values calculated using unpaired two-tailed t-tests of three biological replicates. WT-BRCA2, N=53; WT-BRCA2(+)PARPi, N=54; WT-BRCA2(+)ATRi, N=105; KD-BRCA2, N=107; KD-BRCA2(+)PARPi, N=34; KD-BRCA2(+)ATRi, N=92. Error bars = mean, std. **i** PLA of pSer2-RNAP2 and PCNA in WT- and KD-BRCA2 +/- PARPi or ATRi. Average number of foci per nucleus per biological replicate, *p* values calculated using unpaired two-tailed t-tests of 200 nuclei per condition from 3 biological replicates. Error bars = mean, std. **j** PLA of pSer5-RNAP2 and PCNA in WT- and KD-BRCA2 FTEs +/- PARPi or ATRi. Data presented shows 200 nuclei from 3 biological replicates. *p* values calculated using unpaired two-tailed t-tests. Left: number of foci per nucleus. Right: average number of foci per nucleus per biological replicates. Error bars = mean, std. **k** PLA of pSer2-RNAP2 and PCNA in FTEs +/- 0.2mM HU for 4 hours. *p* values calculated using unpaired two-tailed t-tests of 200 nuclei per condition from 3 biological replicates. Error bars = mean, std.

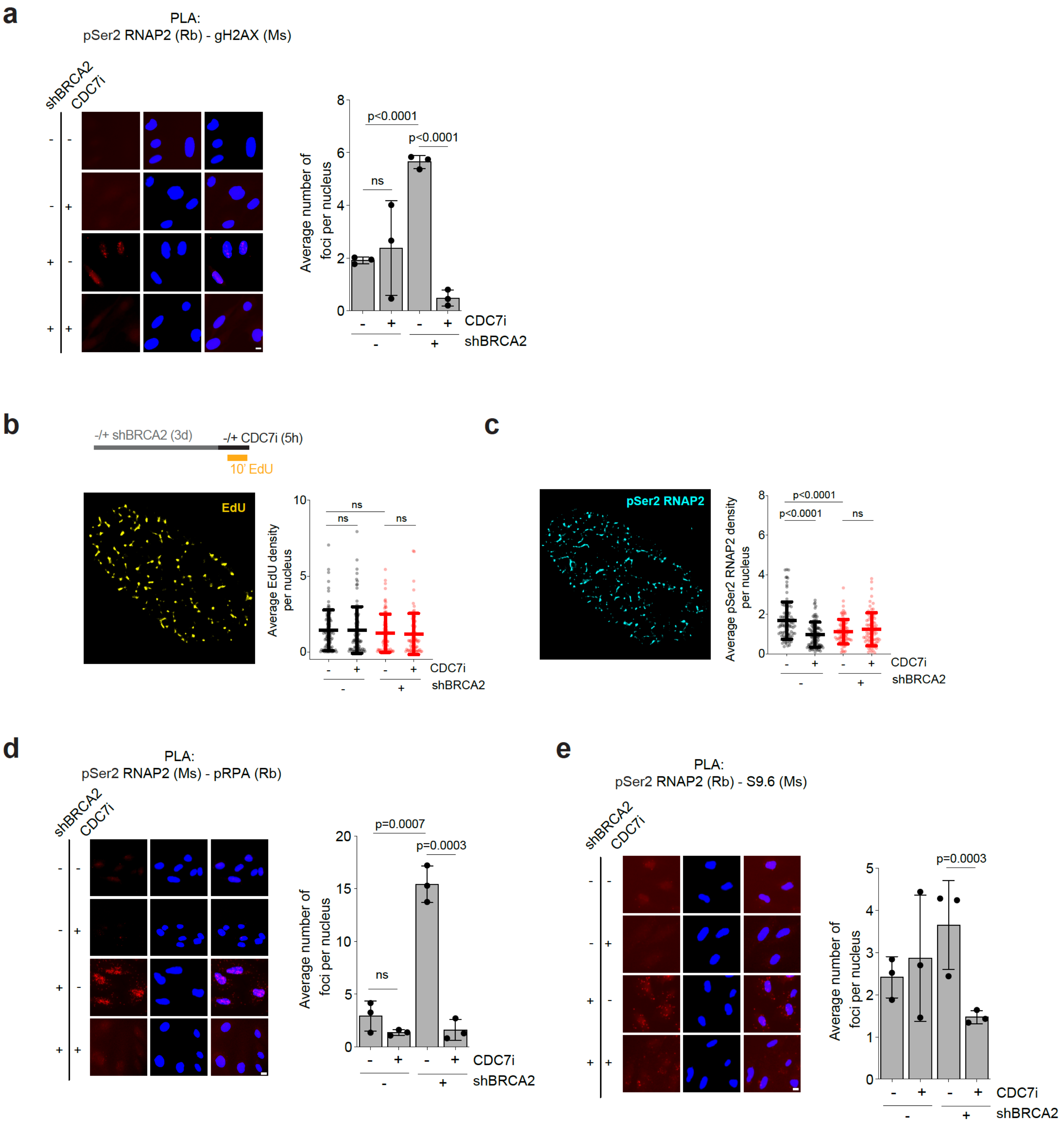

Supplementary Figure 6

Supplementary Fig. 6. HO-TRC-associated genomic instability is rescued by CDC7i treatment. a PLA of pSer2-RNAP2 and gH2AX in WT- and KD-BRCA2 +/-CDC7i including single antibody controls. *p* values calculated using unpaired two-tailed t-tests of 200 nuclei per condition from 3 biological replicates. Error bars = mean, std. Scale bar = 10μm in representative images. **b** Representative SMLM image shows EdU (yellow) of pulse-labeled FTEs +/- shBRCA2 +/- CDC7i. Scatterplot quantification measuring total EdU levels per nucleus. *p* values calculated using unpaired two-tailed t-tests of at least two biological replicates: WT-BRCA2(-)CDC7i, N=91; WT-BRCA2(+)CDC7i, N=96; KD-BRCA2(-)CDC7i, N=83; KD-BRCA2(+)CDC7i, N=75;. Error bars = mean, std. **c** Representative SMLM image shows pSer2 RNAP2 stain (cyan) of FTEs +/- shBRCA2 +/- CDC7i. Scatterplot quantification measuring total EdU levels per nucleus. *p* values calculated using unpaired two-tailed t-tests of at least two biological replicates: WT-BRCA2(-)CDC7i, N=91; WT-BRCA2(+)CDC7i, N=94; KD-BRCA2(-)CDC7i, N=83; KD-BRCA2(+)CDC7i, N=75;. Error bars = mean, std. **d** PLA of pSer2-RNAP2 and pRPA-Ser33 in WT- and KD-BRCA2 +/-CDC7i including single antibody controls. *p* values calculated using unpaired two-tailed t-tests of 200 nuclei per condition from 3 biological replicates. Error bars = mean, std. Scale bar = 10μm in representative pictures. **e** PLA of pSer2-RNAP2 and S9.6 in WT- and KD-BRCA2 +/-CDC7i including single antibody controls. *p* values calculated using unpaired two-tailed t-tests of 200 nuclei per condition from 3 biological replicates. Error bars = mean, std. Scale bar = 10μm in representative images.

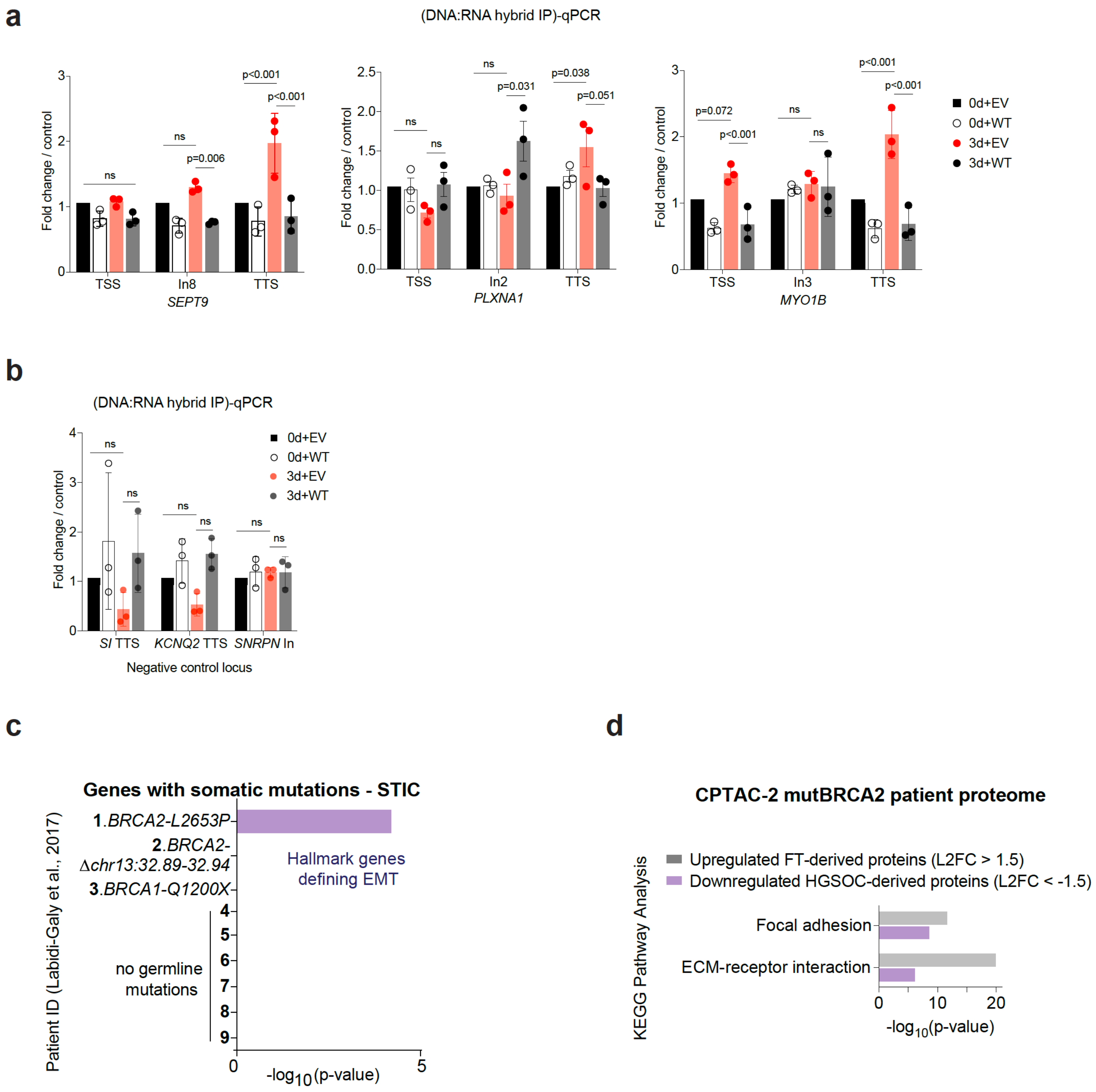

Supplementary Figure 7

Supplementary Fig. 7. HO-TRC-prone genes maintain epithelial integrity in FTEs. a DRIP-qPCR analysis at TSSs and introns of 3 candidate genes in WT- and KD-BRCA2 FTEs +/-RH1 overexpression. Bar graph shows % of input normalized to control sample (0d+EV) of 3 biological replicates. *p* values calculated using two-way ANOVA with Tukey's multiple comparisons test. Error bars = mean, std. **b** DRIP-qPCR analysis at TTSs of 3 low transcriptional volume genes in WT- and KD-BRCA2 FTEs +/-RH1 overexpression. Bar graph shows % of input normalized to control sample (0d+EV) of 3 biological replicates. *p* values calculated using two-way ANOVA with Tukey's multiple comparisons test. Error bars = mean, std. **c** Hallmark GSEA for genes with somatic mutations based on whole exome sequencing from Labidi-Galy et al. (2017) in nine patient samples of STIC lesions and HGSOCs. **d** KEGG pathway enrichment of CPTAC-2 ovarian tumor and normal fallopian tube samples from a mut*BRCA2* patient (McDermott et al. 2020). Data presented shows proteomic pathways significantly upregulated (log_2[fold change]>1.5) in fallopian tube-derived proteome and significantly downregulated (log_2[fold change]<1.5) in HGSOC proteome related to maintenance of epithelium.
