# Supplementary material for: Dormant origin firing promotes head-on transcription-replication conflicts at transcription termination sites in response to BRCA2 deficiency
Source: Nat Commun. 2024 Jun 3;15:4716. doi: 10.1038/s41467-024-48286-1 (PMC11148086; doi:10.1038/s41467-024-48286-1)
Supplement: Supplementary file 3 — Description of Additional Supplementary Files [file 41467_2024_48286_MOESM3_ESM.pdf]

Description of Additional Supplementary Files

File Name: Supplementary Data 1
Description: Table of key resources used for this study
