# Supplementary material for: Dormant origin firing promotes head-on transcription-replication conflicts at transcription termination sites in response to BRCA2 deficiency
Source: Nat Commun. 2024 Jun 3;15:4716. doi: 10.1038/s41467-024-48286-1 (PMC11148086; doi:10.1038/s41467-024-48286-1)
Supplement: Supplementary file 4 — Supplementary Data 1 [file 41467_2024_48286_MOESM4_ESM.pdf]

Supplementary Data 1- Key Resources Table

REAGENT or RESOURCE	SOURCE	IDENTIFIER
Antibodies		
Rabbit polyclonal anti-BRCA2	Abcam	Cat# ab123491, RRID:AB_10972163
Mouse monoclonal anti-BRCA2	Millipore Sigma	Cat#OP95 RRID:AB_2067762
Rabbit polyclonal anti-phospho-RPA32 (ser33)	Thermo	Cat# A300-246A, RRID:AB_2180847
Rabbit polyclonal anti-RPA32	Thermo	Cat# A300-244A, RRID:AB_185548
Mouse monoclonal anti-alpha-Tubulin	Sigma	Cat# CP06, RRID:AB_2617116
Peroxidase-AffiniPure Goat anti-Rabbit IgG (H+L)	Jackson Labs	Cat# 111-035-003, RRID:AB_2313567
Peroxidase-AffiniPure Goat anti-Mouse IgG (H+L)	Jackson Labs	Cat# 115-035-003, RRID:AB_10015289
Rabbit recombinant monoclonal anti-RNA polymerase II CTD repeat YSPTSPS (phospho S2)	Abcam	Cat# ab193468, RRID:AB_2905557
Mouse monoclonal anti-PCNA	Santa Cruz	Cat# sc-56, RRID:AB_628110
Rabbit polyclonal anti-RNA polymerase II CTD repeat YSPTSPS (phospho S5)	Abcam	Cat# ab5131, RRID:AB_449369
Mouse monoclonal anti-RPB1 CTD (4H8)	Cell Signaling	Cat# 2629, RRID:AB_2167468
Rabbit polyclonal anti-PCNA	Thermo	Cat# A300-276A, RRID:AB_263393
Mouse recombinant RNA pol II CTD phospho Ser2	Active Motif	Cat# 91115, RRID:AB_2793780
Mouse recombinant RNA pol II CTD phospho Ser5	Active Motif	Cat# 61086, RRID:AB_2687451
Goat anti-Mouse IgG (H+L) AF488 Conjugated	Thermo	Cat# A-11029, RRID:AB_2534088
Mouse anti-BrdU [B44]	BD Biosciences	Cat# 347580, RRID:AB_10015219
Rat monoclonal anti-BrdU [BU1/75 ICR1)]	Abcam	Cat# ab6326, RRID:AB_305426
Goat anti-Rat IgG (H+L) AF594 Conjugated	Thermo	Cat# A-11007, RRID:AB_10561522
Rabbit monoclonal anti-V5-Tag (D3H8Q)	Cell Signaling	Cat# 13202, RRID:AB_2687461
Rabbit polyclonal anti-RNase H1	Proteintech	Cat# 15606-1-AP, RRID:AB_2238624
Rabbit polyclonal anti-RNase H2A	Thermo	Cat# PA5-20667, RRID:AB_11155195

Rabbit polyclonal anti-BRCA2	Novus	Cat# NBP1-88361, RRID:AB_11036414
Goat anti-Rabbit IgG (H+L) AF647 Conjugated	Thermo	Cat# A-21245, RRID:AB_141775
Rabbit polyclonal anti-RNase H2A	Proteintech	Cat# 16132-1-AP, RRID:AB_2269729
Mouse monoclonal anti-phospho-histone H2A.X (Ser139) [JBW301]	Millipore	Cat# 05-636, RRID:AB_309864
Mouse monoclonal anti-DNA-RNA Hybrid [S9.6]	Kerafast	Cat# ENH001, RRID:AB_2687463
Chemicals, peptides, and recombinant proteins		
Doxycycline	Fisher	BP2653
Hydroxyurea	Sigma	H8267
Biotin TEG-Azide	Berry and Assoc.	BT 1085
CUSO4 – click chemistry grade	Jena Biosciences	M1004-50
Ascorbic acid	Sigma	A92902
Dynabeads MyOne Streptavidin T1	Thermo	65601
cOmplete Mini-Protease Inhibitor Cocktail	Roche	11836170001
5’-ethynyl-2deoxyuridine (EdU)	Sigma	900584
5’-Iodo-2’deoxyuridine (IdU)	Sigma	I7125
5’-chloro-2’deoxyuridine (CldU)	Sigma	C6891
Opti-MEM	Gibco	31985-062
Lipofectamine RNAiMAX	Thermo	13778150
DMEM/F12	Gibco	11320-033
DMEM	Gibco	11995-065
Penicillin-streptomycin	Gibco	15140122
Ultroser G	Sartorius	15950-017
Fetal Bovine Serum	R&D Systems	S11150H
Prolong gold anti-fade reagent	Invitrogen	P36934
Paraformaldehyde 32% solution EM grade	EMS	15714-S
Ultrapure phenol:chloroform:isoamyl alcohol	Thermo	15593049
T4 DNA ligase	Enzymatics	L6030-LC-L
T4 polynucleotide kinase	NEB	M0201L
Proteinase K	Roche	3115828001
Dimethyl sulfoxide (DMSO)	Fisher	BP231-100
Trypsin-EDTA 0.25%	Gibco	25200056
Phusion HF buffer	NEB	B0518S
Phusion high-fidelity DNA polymerase	NEB	M0530
SPRIselect beads	Beckman Coulter	B23317
Fugene HD	Promega	E2311
1-b-D-ribofuanoside	Sigma	SML0350
Formaldehyde 37%	VWR	M134
Protein G Dynabeads	Thermo	10003D
CDC7 inhibitor, PHA767491	Santa Cruz	sc-311303
ATR inhibitor IV, VE-821	Fisher	5-04972-0001

PARP inhibitor, AZD2281	Cayman	10621
Critical commercial assays		
Duolink ® In Situ PLA ® Probe Anti-Rabbit PLUS	Sigma	DUO92002
Duolink ® In Situ PLA ® Probe Anti-Mouse MINUS	Sigma	DUO92004
Duolink ® In Situ Detection Reagents Red	Sigma	DUO92008
Duolink ® In Situ Detection Reagents Green	Sigma	DUO92014
Qubit dsDNA HS assay kit	Thermo	Q32851
KAPA library quantification kit	Roche	KK4824
CellTiter-Glo Luminescent Cell Viability Assay	Promega	G7570
Click-iT Plus EdU Imaging Kit	Thermo	C10646
Universal Mycoplasma Detection Kit	ATCC	301012K
Deposited data		
Raw and processed data – Ok-seq	This study	GEO:GSE239858
Raw and processed data – RNA-seq	This study	GEO:GSE239858
Raw and processed data – Ok-seq	Chen et al. (2019)	GEO:GSE114017
Experimental models: Cell lines		
hTERT FT194	ATCC	CRL3445
hTERT FT194 shScramble	This study	
hTERT FT194 shBRCA2	This study	
Oligonucleotides		
TRIPZ shBRCA2 target sequence	Horizon	TGAGCTTTCGCAACTTC CA
TRIPZ shScramble target sequence	Horizon	ATCTCGCTTGGGCGAGA GTAAG
siRNA – BRCA2#1	Qiagen	5’- CAGCGTTTGTGTATCGG GCAA-3’
siRNA – BRCA2#3	Qiagen	5’- TACGTACTCCA- GAACATTTAA-3’
siRNA – RnaseH2A#4	Qiagen	5’- CAGGTATTCGTGGACAC CGTA-3’
COL5A2 TTS - F	This study	5’- GCATGCCTGAAAACCTG TCA-3’
COL5A2 TTS - R	This study	5’- ACTGCCGTTATCCTTGTC CA-3’
MAP2K2 TTS - F	This study	5’- ACTTTTTGGTGTGTCCG GGG-3’
MAP2K2 TTS - R	This study	5’- CATATGTGCTGGCTTCT GCC-3’
PLXNA1 TTS - F	This study	5’-

		GGTGTGACACATCCCCAAGG-3’
PLXNA1 TTS - R	This study	5’-ACTTACAGGCAGTGCTCAGAC-3’
SEPT9 TTS - F	This study	5’-TGCCAACCTAACTCATTCCAAGA-3’
SEPT9 TTS - R	This study	5’-AGCTCACCTGCAAAGAAAAGC-3’
TOM1L2 TTS – F	This study	5’-TGGGTACTGGTTCTGCCACT-3’
TOM1L2 TTS – R	This study	5’-CAGGGTGGACTCACCAAACC-3’
MYO1B TTS – F	This study	5’ TCACAGCCCTAACTTGTTCCC-3’
MYO1B TTS – R	This study	5’-GGGTCTGGGTACTCCTAGTCA-3’
SEPT9 TSS – F	This study	5’-GCGCAGGGAGAACAAATGAT-3’
SEPT9 TSS – R	This study	5’-GTGCGTCGATTGAGTTGGAG-3’
SEPT9 In8 – F	This study	5’-TAGGGGCTTCCATTCCTCCA-3’
SEPT9 In8 – R	This study	5’-GCTCAGTTCTCCCTTGGTCC-3’
PLXNA1 TSS – F	This study	5’-AAGGGCTCACACTGGGAGAT-3’
PLXNA1 TSS – R	This study	5’-GGCAGAAGAGGGCATGTGAA-3’
PLXNA1 In2 – F	This study	5’-ATCTGTGCTTCCTGGTGAGC-3’
PLXNA1 In2 – R	This study	5’-CCCACAGTGGCCAACTACAT-3’

MYO1B TSS – F	This study	5'-TGTTCAAGGGCCATGTGAGTT-3'
MYO1B TSS – R	This study	5'-GCAATAAACGCTGGGCCGAA-3'
MYO1B In3 – F	This study	5'-AATAGATTGGTGGGGAGTGGGA-3'
MYO1B TSS – R	This study	5'-CAGCTTATGTAGCTACCCCCA-3'
SI TTS – F	This study	5'-TGGTGATCTTCATGACCAGTTGA-3'
SI TTS – R	This study	5'-ACGTATTGATCTGACCACACACA-3'
KCNK2 TTS – F	This study	5'-ATGCTGAGTTTGTGTCCCTC-3'
KCNK2 TTS – R	This study	5'-GTCTGCATGTATTTAGCAAGGGT-3'
SNRPN In – F	Sanz et al. (2019)	5'-GCCAAATGAGTGAGGATGGT-3'
SNRPN In – R	Sanz et al. (2019)	5'-TCCTCTCTGCCTGACTCCAT-3'
Recombinant DNA		
ppyCAG_RNaseH1_WT	Chen et al. (2017)	Addgene #111906
Software and algorithms		
GraphPad Prism v8	https://www.graphpad.com	
ImageJ v1.52a	https://imagej.nih.gov/ij/	
MatLab (v2017b)	https://www.mathworks.com/	
Other		
