# Supplementary material for: Dormant origin firing promotes head-on transcription-replication conflicts at transcription termination sites in response to BRCA2 deficiency
Source: Nat Commun. 2024 Jun 3;15:4716. doi: 10.1038/s41467-024-48286-1 (PMC11148086; doi:10.1038/s41467-024-48286-1)
Supplement: Supplementary file 5 — Reporting Summary [file 41467_2024_48286_MOESM5_ESM.pdf]

nature portfolio

Corresponding author(s): Tony T. Huang

Last updated by author(s): February 29, 2024

Reporting Summary

Nature Portfolio wishes to improve the reproducibility of the work that we publish. This form provides structure for consistency and transparency in reporting. For further information on Nature Portfolio policies, see our Editorial Policies and the Editorial Policy Checklist.

Statistics

For all statistical analyses, confirm that the following items are present in the figure legend, table legend, main text, or Methods section.

n/a	Confirmed	
☐	☒	The exact sample size (*n*) for each experimental group/condition, given as a discrete number and unit of measurement
☐	☒	A statement on whether measurements were taken from distinct samples or whether the same sample was measured repeatedly
☐	☒	The statistical test(s) used AND whether they are one- or two-sided<br>*Only common tests should be described solely by name; describe more complex techniques in the Methods section.*
☒	☐	A description of all covariates tested
☐	☒	A description of any assumptions or corrections, such as tests of normality and adjustment for multiple comparisons
☐	☒	A full description of the statistical parameters including central tendency (e.g. means) or other basic estimates (e.g. regression coefficient) AND variation (e.g. standard deviation) or associated estimates of uncertainty (e.g. confidence intervals)
☐	☒	For null hypothesis testing, the test statistic (e.g. *F*, *t*, *r*) with confidence intervals, effect sizes, degrees of freedom and *P* value noted<br>*Give P values as exact values whenever suitable.*
☒	☐	For Bayesian analysis, information on the choice of priors and Markov chain Monte Carlo settings
☒	☐	For hierarchical and complex designs, identification of the appropriate level for tests and full reporting of outcomes
☒	☐	Estimates of effect sizes (e.g. Cohen's *d*, Pearson's *r*), indicating how they were calculated

Our web collection on statistics for biologists contains articles on many of the points above.

Software and code

Policy information about availability of computer code

Data collection	Super-resolution microscopy data were acquired using Micro-Manager (v1.4)<br>Regular immuno fluroescence data were acquired using Keyence analysis software<br>Sequencing data was acquired using Novaseq 6000 system (Illumina)
Data analysis	Sequencing data were aligned to hg19 using bowtie2, and analyzed using in-house python and R scripts.<br>Codes for Ok-seq analysis are available at https://github.com/FenyoLab/okazaki_origins and https://github.com/FenyoLab/Ok-Seq_Processing. The code is for Research and Educational Purposes for Non-Profit Academic and/or Research Institutions.<br><br>Super-resolution image reconstruction was performed through C++ (via Intel Core i7 7800X) and CUDA8.0 (via NVIDIA GTX 1060) using the Maximum Likelihood Estimation (MLE) algorithm.<br>Codes for Pair-Correlation algorithms, as well as a testing demo (with simulation codes) are available at https://github.com/yiny02/direct-Triple-Correlation-Algorithm. The code is for Research and Educational Purposes for Non-Profit Academic and/or Research Institutions.<br><br>Data analysis software includes ImageJ 1.52a, GraphPad Prism (v8), and Matlab (v2017b)<br>Data presentation/graphing were performed through Matlab (v2017b), GraphPad Prism (v8)<br><br>Code Availability<br>All Ok-seq analysis code is publicly available under the GNU General Public License v2.0 in our GitHub repository: https://github.com/FenyoLab/Ok-Seq_Processing and https://github.com/FenyoLab/okazaki_origins. Code relevant for SMLM analysis is provided here: https://github.com/yiny02/direct-Triple-Correlation-Algorithm. Documentation is provided as a readme file, and specific instructions on parameters

to functions are embedded as inline comments in the code.

For manuscripts utilizing custom algorithms or software that are central to the research but not yet described in published literature, software must be made available to editors and reviewers. We strongly encourage code deposition in a community repository (e.g. GitHub). See the Nature Portfolio guidelines for submitting code & software for further information.

Data

Policy information about availability of data

All manuscripts must include a data availability statement. This statement should provide the following information, where applicable:

- Accession codes, unique identifiers, or web links for publicly available datasets
- A description of any restrictions on data availability
- For clinical datasets or third party data, please ensure that the statement adheres to our policy

Data, including raw sequencing reads and processed data files, is publicly available under GEO accession GSE239858. Scripts are available upon request from the corresponding author and also found in the Github repositories outlined in the software reporting summary herein.

Data Availability
The data supporting the findings of this study are available from the corresponding author upon reasonable request. Further information and requests for resources and reagents should be directed to and will be fulfilled by the lead contact, Tony Huang (tony.huang@nyumc.org). The datasets generated during the current study are available in the NCBI GEO repository under accession number GSE239858. Source data and codes are provided as a Source Data file. Any additional information required to reanalyze the data reported in this paper is available from the lead contact upon request. The results here are in whole or part based upon data generated by the TCGA Research Network: https://www.cancer.gov/tcga. We acknowledge our use of the gene set enrichment analysis, GSEA software, and Molecular Signature Database (MSigDB) (Subramanian, Tamayo, et al. (2005), PNAS 102, 15545-15550, http://www.broad.mit.edu/gsea/).

Research involving human participants, their data, or biological material

Policy information about studies with human participants or human data. See also policy information about sex, gender (identity/presentation), and sexual orientation and race, ethnicity and racism.

Reporting on sex and gender	n/a
Reporting on race, ethnicity, or other socially relevant groupings	n/a
Population characteristics	n/a
Recruitment	n/a
Ethics oversight	n/a

Note that full information on the approval of the study protocol must also be provided in the manuscript.

Field-specific reporting

Please select the one below that is the best fit for your research. If you are not sure, read the appropriate sections before making your selection.

☒ Life sciences ☐ Behavioural & social sciences ☐ Ecological, evolutionary & environmental sciences

For a reference copy of the document with all sections, see nature.com/documents/nr-reporting-summary-flat.pdf

Life sciences study design

All studies must disclose on these points even when the disclosure is negative.

Sample size	For all experiments, sample size was not predetermined, and as much data as possible was collected depending on the nature of the experiments or in order to perform proper statistical analysis, or consistent sample sizes with previously published similar experiments.
Data exclusions	For all super-resolution imaging experiments, EdU(+) S-phase nuclei were selected for analysis, which were then analyzed equally.<br>No other data exclusion was performed
Replication	All biological replicates were performed on separate days.<br>All cell culture experiments were done in independent biological replicates.<br>Two biological replicates were obtained for all Ok-seq samples.<br>All super-resolution experiments were performed at least in duplicate with >60 sample size, as indicated in the manuscript text on separate days.<br>Western blotting experiments were performed in at least three independent experiments.

IF experiments including fibers analysis and PLA were performed in at least three independent experiments.
We followed the same protocols to generate replicates for each of our experiments, and the analysis of the data was reliably reproduced.

Randomization

For all imaging experiments, nuclei on coverslips were randomly selected for imaging across various points on the whole coverslip to correct for differences in staining across the coverslip and between coverslips.
For all super-resolution experiments, illuminated molecules were randomly selected for imaging where bright blinking foci indicated EdU incorporation / S-phase nuclei.

Blinding

For all data and imaging experiments, blinding was not possible as experimental conditions were evident from the imaging data. Image processing and analysis were done using computational pipelines that were applied equally to all conditions and replicates, therefore do not require blinding.

Reporting for specific materials, systems and methods

We require information from authors about some types of materials, experimental systems and methods used in many studies. Here, indicate whether each material, system or method listed is relevant to your study. If you are not sure if a list item applies to your research, read the appropriate section before selecting a response.

Materials & experimental systems

n/a	Involved in the study
☐	☒ Antibodies
☐	☒ Eukaryotic cell lines
☒	☐ Palaeontology and archaeology
☒	☐ Animals and other organisms
☒	☐ Clinical data
☒	☐ Dual use research of concern
☒	☐ Plants

Methods

n/a	Involved in the study
☒	☐ ChIP-seq
☒	☐ Flow cytometry
☒	☐ MRI-based neuroimaging

Antibodies

Antibodies used

Rabbit polyclonal anti-BRCA2 Abcam Cat# ab123491, RRID:AB_10972163; 1/5000 for WB; 1/500 for PLA
Rabbit polyclonal anti-phospho-RPA32 (ser33) Thermo Cat# A300-246A, RRID:AB_2180847; 1/5000 for WB
Rabbit polyclonal anti-RPA32 Thermo Cat# A300-244A, RRID:AB_18554; 1/5000 for WB
Mouse monoclonal anti-alpha-Tubulin Sigma Cat# CP06, RRID:AB_2617116; 1/10000 for WB
Peroxidase-AffiniPure Goat anti-Rabbit IgG (H+L) Jackson Labs Cat# 111-035-003, RRID:AB_2313567; 1/10000 for WB
Peroxidase-AffiniPure Goat anti-Mouse IgG (H+L) Jackson Labs Cat# 115-035-003, RRID:AB_10015289; 1/10000 for WB
Rabbit recombinant monoclonal anti-RNA polymerase II CTD repeat YSPTSPS (phospho S2) Abcam Cat# ab193468, RRID:AB_2905557; 1/500 for PLA
Mouse monoclonal anti-PCNA Santa Cruz Cat# sc-56, RRID:AB_628110; 1/500 for PLA
Rabbit polyclonal anti-RNA polymerase II CTD repeat YSPTSPS (phospho S5) Abcam Cat# ab5131, RRID:AB_449369; 1/500 for PLA
Mouse monoclonal anti-RPB1 CTD (4H8) Cell Signaling Cat# 2629, RRID:AB_2167468; 1/1000 for PLA
Rabbit polyclonal anti-PCNA Thermo Cat# A300-276A, RRID:AB_263393; 1/500 for PLA
Mouse recombinant RNA pol II CTD phospho Ser2 Active Motif Cat# 91115, RRID:AB_2793780; 1/10k for SMLM
Mouse recombinant RNA pol II CTD phospho Ser5 Active Motif Cat# 61086, RRID:AB_26874511; 1/10k for SMLM
Goat anti-Mouse IgG (H+L) AF488 Conjugated Thermo Cat# A-11029, RRID:AB_2534088; 1/5k for SMLM
Mouse anti-BrdU [B44] BD Biosciences Cat# 347580, RRID:AB_10015219; 1/200 for fibers
Rat monoclonal anti-BrdU [BU1/75 ICR1)] Abcam Cat# ab6326, RRID:AB_305426; 1/150 for fibers
Goat anti-Rat IgG (H+L) AF594 Conjugated Thermo Cat# A-11007, RRID:AB_10561522
Rabbit monoclonal anti-V5-Tag (D3H8Q) Cell Signaling Cat# 13202, RRID:AB_2687461; 1/5000 for WB
Rabbit polyclonal anti-RNase H1 Proteintech Cat# 15606-1-AP, RRID:AB_22386241; 1/5000 for WB
Rabbit polyclonal anti-RNase H2A Thermo Cat# PA5-20667, RRID:AB_11155195; 1/5000 for WB; 1/500 for PLA
Rabbit polyclonal anti-BRCA2 Novus Cat# NBP1-88361, RRID:AB_11036414; 1/500 for PLA
Goat anti-Rabbit IgG (H+L) AF647 Conjugated Thermo Cat# A-21245, RRID:AB_141775; 1/300 for fibers
Rabbit polyclonal anti-RNase H2A Proteintech Cat# 16132-1-AP, RRID:AB_2269729
Mouse monoclonal anti-phospho-histone H2A.X (Ser139) [JBW301] Millipore Cat# 05-636, RRID:AB_309864
Rabbit polyclonal anti-BRCA2 Novus Biologicals Cat# NBP1-88361, RRID:AB_11036414
Mouse monoclonal anti-DNA-RNA Hybrid [S9.6] Kerafast Cat# ENH001, RRID:AB_2687463

Validation

https://www.abcam.com/products/primary-antibodies/brca2-antibody-ab123491.html
https://www.thermofisher.com/antibody/product/Phospho-RPA32-Ser33-Antibody-Polyclonal/A300-246A
https://www.thermofisher.com/antibody/product/RPA32-Antibody-Polyclonal/A300-244A
https://www.emdmillipore.com/US/en/product/Anti-Tubulin-Mouse-mAb-DM1A,EMD_BIO-CP06
https://www.jacksonimmuno.com/catalog/products/111-035-003
https://www.jacksonimmuno.com/catalog/products/115-035-003
https://www.abcam.com/products/primary-antibodies/rna-polymerase-ii-ctd-repeat-ysptsps-phospho-s2-antibody-epr18855-ab193468.html
https://www.scbt.com/p/pcna-antibody-pc10
https://www.abcam.com/products/primary-antibodies/rna-polymerase-ii-ctd-repeat-ysptsps-phospho-s5-antibody-ab5131.html
https://www.cellsignal.com/products/primary-antibodies/rpb1-ctd-4h8-mouse-mab/2629

https://www.thermofisher.com/antibody/product/PCNA-Antibody-Polyclonal/A300-276A
https://www.activemotif.com/catalog/details/91115/abflex-rna-pol-ii-ctd-phospho-ser2-antibody-rab
https://www.activemotif.com/catalog/details/61085/rna-pol-ii-ctd-phospho-ser5-antibody-mab
https://www.thermofisher.com/antibody/product/Goat-anti-Mouse-IgG-H-L-Highly-Cross-Adsorbed-Secondary-Antibody-Polyclonal/A-11029
https://www.cellsignal.com/products/primary-antibodies/v5-tag-d3h8q-rabbit-mab/13202
https://www.thermofisher.com/antibody/product/RNASEH1-Antibody-Polyclonal/15606-1-AP
https://www.thermofisher.com/antibody/product/RNAse-H2A-Antibody-Polyclonal/PA5-20667
https://www.novusbio.com/products/brca2-antibody_nbp1-88361

Eukaryotic cell lines

Policy information about cell lines and Sex and Gender in Research

Cell line source(s)	FTE cell lines were obtained as described in the methods (Ronny Drapkin Lab, University of Pennsylvania), and currently available on ATCC: https://www.atcc.org/products/crl-3445<br>Cell lines used are appropriately referenced in the manuscript as well as in the key resources table within the supplementary information file.
Authentication	no cell line authentication was performed or required
Mycoplasma contamination	Cell lines were tested regularly for mycoplasma using Universal Mycoplasma Detection Kit (ATCC)and cell lines used for experiments were those that tested negatively for mycoplasma
Commonly misidentified lines<br>(See ICLAC register)	no commonly misidentified lines were used
